# Effectiveness of SMS Technology on Timely Community Health Worker Follow-Up for Childhood Malnutrition: A Retrospective Cohort Study in sub-Saharan Africa

**DOI:** 10.9745/GHSP-D-16-00290

**Published:** 2018-06-27

**Authors:** Shohinee Sarma, Bennett Nemser, Heather Cole-Lewis, Nadi Kaonga, Joel Negin, Patricia Namakula, Seth Ohemeng-Dapaah, Andrew S. Kanter

**Affiliations:** a Mailman School of Public Health, Columbia University, New York, NY, USA. Now with McMaster University, Hamilton, Canada.; b Millennium Villages Project, Earth Institute, Columbia University. Now with UNICEF, New York, NY, USA. Now with University of the Western Cape, Cape Town, South Africa.; c Yale University School of Epidemiology and Public Health, New Haven, CT, USA. Now with Department of Biomedical Informatics, Columbia University, New York, NY, USA.; d Tufts University School of Medicine, Boston, MA, USA.; e Sydney School of Public Health, University of Sydney, Sydney, Australia.; f Millennium Villages Project, Earth Institute, Columbia University. Now with Columbia Global Centers Africa, Nairobi, Kenya.; g Millennium Villages Project, Earth Institute, Columbia University. Now with Millennium Promise, Dakar, Senegal.; h Millennium Villages Project, Earth Institute, Columbia University, New York, NY, USA. Now with Departments of Biomedical Informatics and Epidemiology, Columbia University, New York, NY, USA.

## Abstract

**Background:**

The Millennium Villages Project facilitated technology-based health interventions in rural under-resourced areas of sub-Saharan Africa. Our study examined whether data entry using SMS compared with paper forms by community health workers (CHWs) led to higher proportion of timely follow-up visits for malnutrition screening in under-5 children in Ghana, Rwanda, Senegal, and Uganda.

**Methods:**

Children under 5 years were screened for malnutrition every 90 days by CHWs using mid-upper arm circumference (MUAC) readings. CHWs used either SMS texts or paper forms to enter MUAC data. Reminder texts were sent at 15 days before follow-up was needed. Chi-square tests assessed proportion of timely follow-up visits within 90 days between SMS and paper groups. Logistic regression analysis was conducted in a step-wise multivariate model. Post-hoc power calculations were conducted to verify strength of associations.

**Results:**

SMS data entry was associated with a higher proportion of timely malnutrition follow-up visits compared with paper forms across all sites. The association was strongest with consistent SMS use over consecutive visits. SMS use at the first of 2 consecutive visits was most effective, highlighting the importance of SMS reminder alerts.

**Conclusions:**

SMS technology with reminders increased timely CHW malnutrition screening visits for under-5 children in Ghana, Rwanda, Senegal, and Uganda, highlighting the importance of such technology for improving health worker behavior in low-resource settings.

## Abstract (precis)

In Ghana, Rwanda, Senegal, and Uganda, we found positive association between community health workers (CHWs) using SMS data entry with reminder alerts and timely follow-up for childhood malnutrition screening visits compared with paper forms. This association was strongest when CHWs used SMS data entry consecutively over multiple visits than when they switched between SMS and paper forms.

## INTRODUCTION

In 2000, the world committed to achieving the measurable targets set by the Millennium Development Goals (MDGs) to combat extreme poverty by 2015. Goal No. 1 targeted halving the proportion of people suffering from hunger globally^1^ and Goal No. 4 aimed to cut the global under-5 mortality rate by two-thirds.^2^ In 2015, more than 99 million children under 5 years of age remained undernourished, with two-thirds of this number in Asia and one-third in Africa.^3^ The pace of progress has not been consistent or equal across regions. The global prevalence of underweight children decreased from 25% (1990) to 15% (2013),^3^ but Africa experienced the smallest relative decrease compared with other regions.^3^ These regional inequalities persist due to complex and multifaceted socioeconomic and political variables. Child health remains a priority in the post-2015 agenda, as evidenced by the targets included in the Sustainable Development Goals (SDGs) 1, 2, and 3 to end poverty, prevent hunger, and improve health, respectively.^4^

The Millennium Villages Project (MVP) was initiated in 2005 to meet these MDGs in rural sub-Saharan Africa with a series of integrated interventions spanning multiple sectors including health, education, agriculture, and infrastructure.^5^ By 2006, MVP operated across 10 countries and 14 community sites in sub-Saharan Africa, covering approximately 500,000 people.^5^^,^^6^ An open-source information and communication system called the Millennium Villages Global Network (MVG-Net) was designed and deployed to assist with health systems monitoring and evaluation and to communicate between sites.^7^

The open-source components of this system used for community health worker (CHW) support were comprised of ChildCount+, a mobile platform used to collect data, and OpenMRS, an electronic medical record in which patient health information was stored.^7^^,^^8^ These 2 components of MVG-Net interacted with each other to collect health information at household visits, store data in a central system, and send CHWs follow-up reminders for pending visits (Figure).^7^ Included in these interventions was the equipping of CHWs with mobile technology (SMS, or short message service) for data collection, reporting, communications, and point-of-care support.

**FIGURE:**
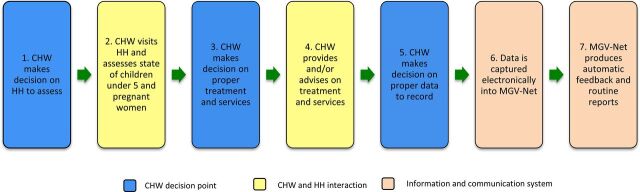
MVG-Net System Framework: Data-Driven Feedback in Real Time to CHWs for Improved Decision Making Abbreviations: CHW, community health worker; HH, households; MVG-Net, Millennium Villages Global Network.

A cluster randomized control trial (RCT) of mobile text message reminders in Kenya evaluated health worker adherence to malaria treatment and counseling.^9^ When health workers received daily text message reminders, there was a 23.7% improvement in correct management immediately and 24.5% improvement at 6 months.^9^ Additional evidence from Western Kenya's MVP site found improved adherence to antenatal care and postpartum visits among CHWs receiving ChildCare+ text reminders.^10^ Similarly, mobile phone use had a positive effect on malaria surveillance, case reporting, and follow-up in rural South Africa.^11^ Quality of SMS reporting by health workers remains a concern; the SMS for Life study across 5 rural Kenyan districts showed variable accuracy of surveillance data.^12^

The field of mHealth has itself burgeoned recently and the use of mobile phones for health interventions has become increasingly sophisticated. The World Health Organization (WHO) describes mHealth as “the practice of medicine and public health assisted by mobile technologies, such as mobile phones, patient monitoring personal digital assistants (PDAs) and other wireless technologies.^13^ In high-income countries, examples include point-of-care monitoring for blood glucose levels in diabetes management^14^ or running a 2-lead electrocardiogram (ECG) for point-of-care cardiac investigations.^15^^,^^16^ In low- and middle-income countries, mobile phones offer a low-cost tool for data collection. The WHO Global Observatory for eHealth (electronic health) reports that the majority (83%) of WHO member states offer at least 1 type of mHealth service within their countries and 70% of mobile phone users reside in low- and middle-income countries.^13^

A systematic review on the use of SMS in health programs identified 31 projects in developing countries in 2012.^17^ The majority of these programs were in Africa (Kenya and South Africa) followed by Asia (India). Within Africa, programs were concentrated in the Great Lakes region, particularly Kenya, Tanzania, and Uganda, where the mobile phone market has recently expanded.^17^ The purpose of these interventions included disease prevention, surveillance, self-management, treatment adherence,^17^ and improved health worker behavior.^18^ Despite this interest, academic research in the field is minimal and mixed,^19^^,^^20^ particularly in Africa.^21^ Studies are mainly single observational data points rather than multinational or regional research.^22^

The purpose of this article is to examine whether SMS patient data entry with text message reminders influenced CHW follow-up visits compared with paper data entry. Our study uniquely shows evidence from multiple MVP country sites across sub-Saharan Africa.

The purpose of this article is to examine whether SMS patient data entry with text message reminders influenced CHW follow-up visits compared with paper data entry.

## INTERVENTION

The MVG-Net system's purpose was to gather health information in order to provide a feedback mechanism to inform patient management.^7^ Standard mobile phones with SIM cards were provided to CHWs.^8^ These phones included the ChildCount+ platform, an open-source system built on Django web framework and Python language.^8^ CHWs used these phones to send SMS formatted texts to register patients during home visits and enter their individual and household data into a central database.^8^ Household heads, children under 5 years, and all pregnant women in a household were registered using a unique patient identifier (Patient ID) with a linked household identifier (Household Head ID) at the first home visit. Malnutrition screening for children under 5 years occurred at 90-day intervals.^23^

After performing a nutritional screen that included checking mid-upper arm circumference (MUAC) and signs of edema for all under-5 children in a household, CHWs sent a formatted SMS text message into the ChildCount+ system.^8^ The ChildCount+ system then sent a reminder text message to the CHW's phone 75 days after the previous MUAC screen.^23^ If the CHW did not conduct a follow-up MUAC screen within 90 days, then the system would send weekly text reminders until follow-up was achieved.^23^ CHWs using paper forms for data entry did not receive these reminder alerts. CHWs were also responsible for ad hoc visits to follow-up if a child was malnourished or after a recent discharge from clinic. These ad hoc visits were prioritized over regular follow-ups when needed.

### CHW Training

Integral to MVP's focus on health systems was integrated community case management. CHWs played a large role in regular home visits and the patient referral process. CHWs were generally chosen by the communities and given initial training on community health including maternal and child health.^24^ Scope of practice involved clear tasks during household visits including registering individuals, identifying sick individuals, following up on young children and pregnant women, and reporting visits to CHW supervisors. A defined management structure with regular remuneration was in place, including necessary resources such as medical supplies, mobile phones, and bicycles, among others.^24^ As per the Earth Institute's *Technical Task Force Report* on CHW scale-up and management, the average estimated cost of funding CHWs per head per year for the covered rural population was $6.56.^25^

CHWs scheduled a total of 30 home visits per week with 5 visits per day over a 6-day work week. These visits included a mix of follow-up and regular visits, which prioritized pregnant women and infants. Sick individuals were referred to the nearby primary health care facility. At the end of each month, weekly logbooks outlining each visit were presented to CHW supervisors. The supervisor analyzed visit history and redistributed caseloads as necessary. CHW supervisors were generally experienced senior CHWs with supervisory training, who then reported to CHW managers at the primary health care facility. There was an approximate minimum ratio of 150 households per CHW across MVP sites.^25^

### Implementation Challenges

There were 2 components to data collection involved in this study: CHWs entered patient and household data and tracked their home visits during an initial 3- to 6-month period using only paper forms followed by 3 to 6 months of using only SMS entries. During the transition from paper forms to mobile phones, data collection was validated using both paper and SMS for at least a 1-month period in order to ensure operational effectiveness and data accuracy. The date of visit on the paper forms was the actual date visited, whereas the date of visit on SMS entries was not entered but assumed to be the date of SMS transmission. There were no major server outages or SMS coverage disturbances during this study period that could have offset consistent reporting.

For 3 to 6 months, CHWs entered data using only paper forms, followed by using only SMS entry for another 3 to 6 months but some CHWs switched between paper and SMS for the same patients.

Variations in implementation did occur across sites and was likely due to unfamiliarity with the tool or CHW-related factors. These variations allowed for concomitant use of paper forms and SMS data entry outside the prescribed overlap period. CHWs switched between paper and SMS entries for follow-up visits for the same patients. This switching created 4 comparison groups for evaluation over consecutive visits:

1. SMS entry at initial visit followed by paper form at follow-up visit
2. Paper form at initial visit followed by SMS entry at follow-up visit
3. SMS entries at both visits
4. Paper forms at both visits

When CHWs used SMS at the first of 2 consecutive visits (groups 1 and 3), a reminder text would still be sent after 75 days. This text would not be sent when using paper forms at the first visit (groups 2 and 4).

## METHODS

### Study Design

The study design is a retrospective observational study with follow-up over 30 months, from February 1, 2010, through July 31, 2012. The 2 comparison groups were SMS for data entry versus paper forms.

### Study Sites

Four MVP sites were selected for this study: Ruhiira (Uganda), Bonsaaso (Ghana), Potou (Senegal), and Mayange (Rwanda). These sites were chosen based on the continuity of accurate data available over the study period. These sites represent a collection of villages with a total catchment population of approximately 155,740 people.^6^ Ruhiira is the largest of the 4 sites with 8 villages and more than 10,000 households (Table 1). Bonsaaso is a group of 6 villages with about 5,700 households. Potou, similarly, has 6 villages with approximately 3,200 households. Lastly, Mayange is made up of 4 villages with about 5,500 households.

**TABLE 1.** Snapshot of MVP Sites Catchment Data^a^

Site	No. of Villages	Population	No. of Households	No. of Health Centers
Ruhiira, Uganda	8	50,000	10,385	6
Bonsaaso, Ghana	6	35,000	5769	7
Potou, Senegal	6	32,000	3249	5
Mayange, Rwanda	4	23,000	5500	1

Abbreviation: MVP, Millennium Villages Project.

a Catchment data represent baseline data from the MVP midterm analysis conducted in 2009.

### Study Population and Inclusion Criteria

The study population across the 4 sites included children aged 6–60 months^26^ who received an MUAC screen from a CHW between February 1, 2010, and July 31, 2012. Only patient data entries that could be linked to MUAC readings were included. Participants with missing MUAC readings or missing information on type of data entry (SMS or paper) were excluded. Children with MUAC screens who turned 5 during the course of follow-up were included. Children under 6 months of age were excluded because they do not receive regular MUAC screens as per WHO growth monitoring standards.^27^

### Study Follow-Up Period

Appropriate follow-up by CHWs was defined as an MUAC reading within 90 days from previous measurement (during the relevant age range). Prior research demonstrated that MUAC measurements were independently comparable predictors of child mortality compared with weight-for-age *z* score indices.^28^ Risk of childhood mortality was shown to significantly increase when MUAC measurements fell below 115 mm and it is one of the WHO diagnostic criteria for severe acute malnutrition.^26^^,^^29^ The 90 days maximum for follow-up MUAC readings was chosen since MUAC measurements were previously shown to predict mortality within 30- and 90-day intervals.^30^

### Variables

#### Exposure Variable

The exposure variable was a dichotomous variable representing MUAC data entries using either SMS (1) or paper forms (0) for children aged 6–60 months over the entire study period.

#### Outcome Variable

The outcome variable captured each follow-up MUAC entry after the first MUAC entry for a unique child. The outcome variable was coded as a dichotomous variable: proportion of follow-up MUAC entries within 90 days (1) or after 90 days (0).

#### Covariates

Six explanatory variables were selected *a priori* and included age of child, gender of child, age of household head, gender of household head, number of children per household, and number of children per CHW. Each of these covariates was created as a continuous variable initially. In order to assess subgroup differences, the variables were then transformed into categorical variables.

### Data Selection

The method of selecting study participants was non-randomized; there was no participant sampling because the entire study site was evaluated. All children between 6–60 months who received a household visit between February 1, 2010, and July 31, 2012, and an MUAC assessment at any point in this duration were automatically enrolled.

### Power and Sample Size Calculation

We conducted post-hoc power and sample size calculations to guide interpretation of our findings (Supplement 1).^31^ We used 5% alpha level and a 2-sample comparison of proportion of timely follow-up visits between data and SMS entry.

### Data Analysis

Household-level data were reformatted and merged to patient-level data by matching Household Head IDs with Patient IDs in order to group patients who belonged to a unique household together. Duplicates were removed from each variable separately before merging all observations together by Patient ID.

Chi-square tests at 5% significance level were conducted between exposure and outcome variables, and also between all covariates and exposure and outcome variables. Logistic regression was used to examine the strength of the association between exposure and outcome in both crude and adjusted models. All covariates were included in the multivariate regression models regardless of associated *P* values. The final adjusted multivariate model included analysis of the switch between paper and SMS entries to elicit whether using SMS even at 1 visit had a benefit in timely follow-up due to the 75-day text reminder. This relationship between type of data entry switching and proportion of timely visits was coded as following:

(0) Paper forms over 2 consecutive visits
(1) SMS at first visit, paper forms at second visit
(2) Paper forms at first visit, SMS at second visit
(3) Only SMS over 2 consecutive visits

All statistical tests were conducted using STATA v.10 (STATACorp, Texas, USA).

### Patient Confidentiality and Ethics

All MVG-Net personnel received training on patient confidentiality and data access. Only health care providers had access to primary identified data. The data were de-identified and stored in the OpenMRS system using secure, confidential, password-protected means. The de-identified data were retrospectively accessed by the research analysis team at the Earth Institute at Columbia University. All de-identified retrospective raw data are available in Supplements 2–5.

This study was approved by the Columbia University Institutional Review Board (IRB) under protocol number IRB AAAF1647.

## RESULTS

### Descriptive Statistics

The number of children with MUAC data entries ranged across the study sites from 1,970 in Ghana, to 10,256 in Uganda (Table 2). The median number of under-5 children per household was 1 in Ghana and Rwanda, and 2 in Uganda and Senegal. CHWs in Uganda and Senegal saw more children (median of 177 and 162, respectively) than CHWs in Ghana and Rwanda (median of 60 and 35, respectively).

**TABLE 2.** Descriptive Statistics by Study Site, 2010–2012

	Bonsaaso, Ghana	Ruhiira, Uganda	Mayange, Rwanda	Potou, Senegal
**Under-5 Children**	N=2563	N=13,404	N=2398	N=5765
No. of children (6–60 months) with MUAC data entries	N=1970	N=10,256	N=2250	N=5038
Median age of under-5 children, years (IQR)	2.1 (2.5)	2.3 (2.3)	2.9 (2.5)	2.5 (2.5)
Age groups for under-5 children, months, No. (%)				
0–6^a^	310 (14)	1860 (14)	238 (10)	631 (12)
6–12	283 (13)	1442 (11)	286 (12)	599 (11)
12–18	235 (11)	1308 (10)	147 (6)	557 (11)
18–24	272 (13)	1190 (9)	216 (9)	563 (11)
24–30	262 (12)	1262 (9)	261 (11)	631 (12)
30–36	208 (10)	1125 (8)	246 (11)	520 (10)
36–42	212 (10)	1241 (9)	342 (15)	557 (11)
42–48	171 (8)	1157 (9)	248 (11)	528 (10)
48–54	189 (9)	1443 (11)	325 (14)	651 (12)
54–60	–	1444 (10)	–	–
Gender of under-5 children, No. (%)				
Male	1250 (49)	6643 (50)	1247 (52)	2918 (51)
Female	1313 (51)	6761 (50)	1151 (48)	2847 (49)
**Households**	**N=4956**	**N=11,703**	**N=4631**	**N=4146**
Median age of household head, years (IQR)	35.5 (14.3)	32.0 (13.0)	33.0 (12.0)	40.5 (15.8)
Median number of under-5 children in each household (IQR)	1 (1.0)	2 (1.0)	1 (1.0)	2 (1.0)
Gender of household head, No. (%)				
Male	3900 (79)	8529 (73)	3467 (75)	3980 (96)
Female	1056 (21)	3174 (27)	1164 (25)	166 (4)
**Community Health Workers**	**N=78**	**N=70**	**N=155**	**N=40**
Median number of under-5 children per community health worker (IQR)	60 (63)	177 (50)	35 (35)	162 (107)

Abbreviations: IQR, interquartile range; MUAC, mid-upper arm circumference.

^a^ Not included in analysis as MUAC readings are conducted in children ages >6 months and <5 years.

### Bivariate Analyses

Two-sided bivariate analyses with chi-square tests of SMS versus paper entry including all covariates was conducted at 5% level of significance (Table 3). All covariates had a statistically significant association with the outcome except gender of the child and of the household head.

**TABLE 3.** Chi-Square Analysis of Paper Versus SMS Follow-Up by Study Population Characteristics

Characteristic	Uganda		Ghana		Senegal		Rwanda	
	MUAC >90 days (%)	*P* Value	MUAC >90 days (%)	*P* Value	MUAC >90 days (%)	*P* Value	MUAC >90 days (%)	*P* Value
**Under-5 Children**								
Age, months		<.001***		<.001***		<.001***		<.001***
6–12 (1 year)	64	REF	67	REF	26	<.001***	64	.11
12–18	70	<.001***	80	<.001***	34	<.001***	79	.001***
18–24 (2 years)	72	<.001***	77	<.001***	36	<.001***	76	.03*
24–30	72	<.001***	70	<.001***	36	<.001***	69	.96
30–36 (3 years)	73	<.001***	77	<.001***	32	<.001***	75	REF
36–42	73	<.001***	80	<.001***	35	<.001***	68	.67
42–48 (4 years)	74	<.001***	73	<.001***	33	<.001***	78	.002***
48–54	71	<.001***	71	<.001***	30	<.001***	75	.045*
54–60 (5 years)	62	<.001***	62	<.001***	20	.30	76	.02*
Gender		.006**		**.25**		**.98**		**.76**
Male	70	.006**	73	REF	32	REF	73	REF
Female	71	REF	74	.25	32	.98	74	.76
**Households**								
No. of under-5 children in each household categorized		<.001***		.002**		**.05** *		**.047** *
1	56	REF	72	REF	33	REF	74	REF
2	60	.03*	76	<.001***	33	.94	74	.75
3	60	<.001***	78	.001***	31	.16	71	.61
4	61	.63	72	.88	31	.33	63	.20
5	71	.03*	30	.01**	32	.67	11	.003**
6	68	.37	NA	NA	30	.28	NA	NA
7	NA	NA	NA	NA	14	.04*	NA	NA
8	NA	NA	NA	NA	41	.20	NA	NA
9	NA	NA	NA	NA	40	.28	NA	NA
10	NA	NA	NA	NA	16	.09	NA	NA
11	NA	NA	NA	NA	NA	NA	NA	NA
Age groups for household heads, years		<.001***		<.001***		**.82**		**.02** *
0–15	66	.11	67	.30	NA	NA	NA	NA
15–25	48	<.001***	41	<.001***	27	REF	NA	NA
25–35	56	REF	55	REF	26	.49	54	<.001***
35–45	59	<.001***	54	.20	28	.74	67	REF
45–55	59	<.001***	61	<.001***	27	.98	68	.45
55–65	60	<.001***	58	.33	26	.50	66	.80
≥65	60	<.001***	55	.99	26	.61	70	.31
Gender of household head		**.05** *		**.88**			**.26**	<.001***
Male	56	REF	55	REF	27	REF	69	REF
Female	57	.05*	54	.88	29	.26	54	<.001***
**CHWs**								
No. of under-5 children per CHW		<.001***		<.001***		<.001***		<.001***
1 (range: 0–150)	71	REF	76	REF	NA	REF	64	REF
2 (range: 150–177)	72	.56	77	.66	NA	NA	70	<.001***
3 (range: 177–200)	72	.005**	81	.002**	17	.45	80	<.001***
4 (range: ≥200)	67	<.001***	63	<.001***	32	<.001***	NA	NA

Abbreviations: CHW, community health worker; MUAC, mid-upper arm circumference; NA, not applicable.

* *P*≤.05;

** *P*≤.01;

*** *P*≤.001.

The percentage of follow-up visits occurring within 90 days by SMS entry and paper forms is displayed in Table 4. In all sites, a greater proportion of follow-up visits occurred within 90 days when CHWs entered data through SMS versus paper forms. For example, in Uganda 92% of follow-up visits occurred within 90 days when data were entered through SMS compared with 78% when data were entered using paper forms. Similarly, in Ghana the percentages were 85% and 46%, respectively. Associated chi-square tests demonstrated *P* values <.001 across all sites.

**TABLE 4.** MUAC Follow-Up Within and After 90 Days by Paper Entry Versus SMS Entry and Reminders

Country	No. (%)	
	Paper Entry	SMS Entry + Reminders
Uganda		
≤90 days	67,374 (78)	9,885 (92)
>90 days	18,796 (22)	914 (8)
Ghana		
≤90 days	487 (46)	5,123 (85)
>90 days	583 (54)	921 (15)
Senegal		
≤90 days	1,248 (22)	3,576 (40)
>90 days	4,509 (78)	5,376 (60)
Rwanda		
≤90 days	1,253 (59)	1,651 (92)
>90 days	869 (41)	135 (8)

Abbreviations: MUAC, mid-upper arm circumference; SMS, short message service.

Chi-square tests demonstrated *P* values <.001 across all sites.

### Multivariate Analyses

#### Ruhiira, Uganda

At the Uganda site, there was a strong positive crude association between SMS data entry and timely MUAC follow-up visits within 90 days of previous visit (odds ratio [OR]=3.02; 95% confidence interval [CI]: 2.82, 3.24) compared with paper entry. This association was stronger after adjusting for confounding in the logistic regression analysis (OR=3.23; 95% CI: 2.90, 3.59). Compared with only paper entries as a reference, the adjusted association was strongest with consistent SMS use over consecutive visits (OR=18.14; 95% CI: 12.99, 25.32) (Table 5). The adjusted association was significantly weaker when paper entry was used at the first and SMS entry at the second of 2 consecutive visits (OR=1.98; 95% CI: 1.81, 2.15), whereas the strength of the association remained when using SMS first across 2 consecutive visits (OR=3.23; 95% CI: 2.91, 3.60).

**TABLE 5.** Association Between Timely Follow-up and Switching Between SMS and Paper Forms Over 2 Consecutive Visits (Multivariate Model)

SMS-Paper Switching	Uganda		Ghana		Senegal		Rwanda	
	Crude OR (95% CI)	Adjusted OR (95% CI)	Crude OR (95% CI)	Adjusted OR (95% CI)	Crude OR (95% CI)	Adjusted OR (95% CI)	Crude OR (95% CI)	Adjusted OR (95% CI)
Paper-Paper (0 0)	REF	REF	REF	REF	REF	REF	REF	REF
Paper-SMS (0 1)	2.04 (1.88, 2.23)	1.98 (1.81, 2.15)	0.16 (0.12, 0.22)	0.17 (0.12, 0.23)	0.24 (0.21, 0.27)	0.25 (0.22, 0.29)	1.30 (1.02, 1.65)	1.31 (1.02, 1.67)
SMS-Paper (1 0)	3.34 (3.01, 3.71)	3.23 (2.91, 3.60)	2.52 (0.58, 10.97)	2.02 (0.44, 9.34)	1.91 (1.63, 2.23)	2.00 (1.71, 2.35)	5.43 (3.14, 9.40)	5.33 (3.06, 9.27)
SMS-SMS (1 1)	18.54 (13.34, 25.76)	18.14 (12.99, 25.32)	2.99 (2.34, 3.83)	3.01 (2.32, 3.91)	1.38 (1.24, 1.54)	1.43 (1.28, 1.60)	10.52 (7.39, 14.97)	9.89 (6.91, 14.14)

Abbreviations: CI, confidence interval; OR, odds ratio; SMS, short message service.

#### Bonsaaso, Ghana

At the Ghana site, the crude association between using SMS entry versus paper entry and MUAC follow-up visits within 90 days was strongly positive (OR=6.66; 95% CI: 5.79, 7.65). However, when adjusted for confounding, this association was not significant (OR=1.78; 95% CI: 0.39, 8.04). The adjusted association did not become statistically significant regardless of SMS entry at the first consecutive visit (OR=2.02; 95% CI: 0.44, 9.34) or at the second consecutive visit (OR=0.17; 95% CI: 0.12, 0.23) (Table 5). There was, however, a statistically significant adjusted association between SMS entry over consecutive visits and timely MUAC follow-up (OR=3.01; 95% CI: 2.32, 3.91).

#### Potou, Senegal

The Senegal site also had positive crude (OR=2.40; 95% CI: 2.23, 2.59) and adjusted (OR=2.03; 95% CI: 1.73, 2.38) associations between SMS data entry and timely MUAC follow-up within 90 days compared with paper entry. Similar to Uganda and Ghana, the adjusted dose-dependent association was stronger when SMS was used at the first consecutive visit followed by paper entry at the second visit (OR=2.00; 95% CI 1.71, 2.35) (Table 5). There was a negative association between using paper entry at the first visit and SMS at the second consecutive visit (OR=0.25; 95% CI: 0.22, 0.29). Consistent SMS use over consecutive visits had an improved association with timely follow-up (OR=1.43; 95% CI: 1.28, 1.60), but not as strong as the Uganda and Ghana sites.

#### Mayange, Rwanda

Between all study sites, the crude association between SMS versus paper entry and timely 90-day MUAC follow-up was the strongest for Rwanda (OR=8.48; 95% CI: 6.97, 10.31). There was still a strong positive association when adjusted for confounding (OR=5.09; 95% CI: 2.92, 8.87). The adjusted association grew stronger with SMS use at the first visit (OR=5.33; 95% CI: 3.06, 9.27) compared with paper entry at the first visit (OR=1.31; 95% CI: 1.02, 1.67) (Table 5). The adjusted association was strongest with consistent SMS use over consecutive visits (OR=9.89; 95% CI: 6.91, 14.14).

Between all 4 study sites, the association between SMS vs. paper entry and timely follow-up was strongest for Rwanda.

## DISCUSSION

The results of our study show a clear positive association between SMS data entry with reminder alerts and timely CHW follow-up for malnutrition screening visits. This finding is consistent with other studies that show improvement in case reporting, follow-up, and treatment by CHWs using SMS text reminders. Our study focused on the process indicator of proportion of timely malnutrition follow-ups using SMS versus paper data entry. This analysis does not comment on what program evaluation literature describes as outcome or impact indicators to describe adequacy of child nutritional support.^32^ Mitchell et al. recently published the endline evaluation of 40 outcomes across 10 MVP-scaled sites in categories including nutrition and child health.^5^ We refer readers to this evaluation for end outcomes that are overall positive for the study duration.

Across all study sites, the association with timely follow-up was strongest when SMS data entry was used consecutively over multiple visits compared with switching between SMS and paper entry. Using SMS entry at the first of 2 consecutive visits still showed benefits, likely due to the reminder alerts sent at 75 days. The poorer results in some sites are difficult to explain without qualitative field data. Future qualitative research including interviews with CHWs and site administrative staff would help to explain differences in SMS implementation. This knowledge would help improve implementation in the future and prevent switching between SMS and paper entry. Our analysis reflects what occurred on the ground across sites during program implementation and shows benefit of using SMS consistently over follow-up visits.

Across all study sites, the association with timely follow-up was strongest when SMS data entry was used consecutively than when CHWs switched between SMS and paper.

Additionally, SMS use may be largely dependent on individual CHW characteristics and patient demographics. In our study, CHWs were more likely to use SMS entry when there were more than 200 children per CHW (Senegal and Uganda). Similarly, other factors may influence CHW adherence to mobile phones including CHW age, gender, and training duration. Future analyses could explore patient and CHW characteristics associated with SMS use behavior.

### Limitations

According to post-hoc power calculations, the study was highly powered across all 4 sites (Supplement 1), which could reflect the large effect sizes. A multilevel regression model combining all 4 sites was initially considered, but this analysis was underpowered and difficult to achieve due to variations in site implementation. The lack of a clear comparison group in this study makes it difficult to control for unknown factors that influence patient follow-up. Without qualitative data, it is difficult to know why CHWs sometimes switched between paper and SMS data entry. This switching could occur due to various reasons such as poor cellular reception, short battery life, or lack of adequate training. More qualitative data about the study sites may help understand why Uganda had such a low proportion of SMS use compared with Ghana, Rwanda, and Senegal. There were no reported power outages or natural reasons causing poor SMS use in Uganda, but it is possible that an administrative challenge led to disruptions in service. Delays in follow-up visits could also be related to difficult and hilly terrains specifically in Ruhiira, Uganda, impacting both SMS and paper entry groups.

## CONCLUSION

Using SMS for patient data entry with reminder alerts led to more timely CHW follow-up visits for malnutrition screening across multiple countries. The study highlights the importance of SMS technology in improving community-based health care delivery in low-income countries.

## Supplementary Material

16-00290-Sarma-Supplement5-Rwanda.xlsx

16-00290-Sarma-Supplement2-Uganda.xlsx

16-00290-Sarma-Supplement1.xlsx

16-00290-Sarma-Supplement3-Ghana.xlsx

16-00290-Sarma-Supplement4-Senegal.xlsx

## Notes

**Peer Reviewed**

**Funding:** The Novartis Foundation for Sustainable Development supported this research.
